# Editorial: Colorectal surgery and proctology: past, present, and future

**DOI:** 10.3389/fsurg.2024.1373867

**Published:** 2024-02-01

**Authors:** Francesco Pata, Roberta Tutino, Arcangelo Picciariello, Francesco Cantarella

**Affiliations:** ^1^ Department of Pharmacy, Health and Nutritional Sciences, University of Calabria, Rende, Italy; ^2^ Department of Surgery, AO Annunziata, Cosenza, Italy; ^3^ Department of General and Emergency Surgery, AOU Città Della Salute e Della Scienza di Torino, Turin, Italy; ^4^ Department of Experimental Medicine, University of Salento, Lecce, Italy; ^5^ CPEP (Centre for Proctology and Perineology), Ospedali Privati Forlì, Forlì, Italy

**Keywords:** colorectal, emerging techniques, surgery, haemorrhoids, robotics, hemorrhoids, sclerobanding, anticoagulant

**Editorial on the Research Topic** Colorectal surgery and proctology: past, present, and future

“*The patient is the center of the medical universe around which all our works revolve and towards which all our efforts tend*” John Benjamin Murphy (1857–1916) (1).

Colorectal surgery encompasses a wide array of interventions, ranging from the management of functional disorders to the treatment of oncological conditions. This field includes pathologies amenable to cost-effective, office-based approaches (2–4), as well as complex diseases requiring a multidisciplinary strategy and advanced, expensive procedures such as robotic surgery (5), and innovative technologies driven by artificial intelligence and machine learning (6). However, further evidence is needed in many areas, as some key aspects of decision-making still rely on low-quality studies and/or expert opinion.

In this Research Topic, contributors have concentrated on several highly debated scenarios. In the proctology field, attention was directed towards unresolved aspects of hemorrhoid management under specific conditions, along with the atypical manifestations of anal fistulas. Regarding the colorectal field, investigations included the application of indocyanine green (ICG) in laparoscopic surgery, the persistent challenges in diagnosing and preventing anastomotic leaks, and other postoperative complications such as surgical site infections.

High-risk patients with bleeding hemorrhoids on anticoagulation therapy or antiplatelet drugs represent a challenging group in clinical practice. Whether to suspend or continue anticoagulant therapy during the perioperative period is still a controversial issue (7). Pata et al., in their pilot study, reported the preliminary results of sclerobanding (8, 9), a new office-based technique, performed on 51 anticoagulated patients without suspending therapy. They noted only minor postoperative complications and no cases of readmission or mortality. While further studies on a larger patient cohort are necessary, these promising results pave the way to a tailored approach for this subgroup of patients.

Currently, anastomotic leak is the most feared complication following colorectal resections (10, 11), leading to increased postoperative complications, mortality, and potentially detrimental effects on long-term survival (12). Ozata et al. tested a novel scoring system, the bedside leak score, on a cohort of 184 patients. This score, obtained by dividing the CRP quotient (third postoperative day CRP level/first postoperative CRP level) by the preoperative albumin level, demonstrated better performance in AL detection compared to other scores, with 90.9% sensitivity and 59.3% specificity at an optimal cut-off value of 50.3. Given the widespread availability and reproducibility of its variables, this scoring system, if validated further, could significantly improve early diagnosis and treatment of AL, benefiting a large patient cohort.

Surgical site infections (SSIs) continue to be a frequent complication in colorectal surgery with deleterious impacts on patients, healthcare system, and community (13, 14), with an increased postoperative mortality. Sun et al. analyzed risk factors associated with SSIs, noting a significant reduction in SSIs rates post-colorectal surgery, from 13.33% in 2010 to 3.56% in 2019 (*p* < 0.001) when measures such as correcting preoperative hypoproteinemia, choosing laparoscopic surgery and preoperative bowel preparation were adopted. These data are worthy of a mention as proof of how it is possible to significatively reduce SSI rate, that reaches 30% in some studies (15).

The introduction of ICG has been a game-changer in digestive surgery (16). Its preoperative or intraoperative infusion can improve primary tumor or metastasis detection, as well as the identification of lymphatic pathways and sentinel lymph nodes (SLN) (17, 18), especially in laparoscopic and robotic surgeries, where the lack of tactile sensation can make tumor identification more challenging.

Konstantinidis et al. performed a systematic review on preoperative tumour tattooing with ICG before minimally invasive colorectal surgery, including 696 patients from eight single-centre studies. They demonstrated a high detection rate (97%) when surgery was performed within a week of injection, with no evidence of ICG-related complications.

Advancements in artificial intelligence and machine learning are poised to support researchers and policymakers in better interpreting data and developing predictive models to assist clinicians in evidence-driven decision-making (19). Lu et al. advanced in this direction, proposing a web-based predictive model including 9 preoperative variables, to discriminate between localized colorectal cancer and colorectal adenoma. Despite some methodological limitations and the need for validation with multicentre prospective study data, this approach is likely to benefit from emerging technologies in the next future.

Understanding risk factors that can affect postoperative outcomes is crucial for a tailored surgery. Jiang et al.’s propensity score matching analysis showed that chronic liver disease (CLD) significantly increases postoperative complications and length of stay in patients undergoing simultaneous resection of colorectal cancer and liver metastases compared to patients without CLD.

Furthermore, unusual presentations of common diseases can lead to suboptimal or delayed treatment, resulting in adverse outcomes for patients. Zhou et al. highlighted this issue in cases of retroperitoneal abscess as the first presentation of colon cancer. Their analysis of 61 patients showed that these patients often underwent multiple unnecessary treatments with high mortality, emphasizing the need for prompt recognition and treatment.

A potential counterpart in proctology might be anal fistulas with scrotal extension. Vo et al. examined 150 patients with this condition, analyzing MRI and intraoperative features. They found a high correlation between MRI and intraoperative data, with many of these fistulas exhibiting an anterior internal orifice and a long, low-transsphincteric tract. This information adds important insights to current clinical practice.

Like other areas of surgery, colorectal surgery still harbors numerous questions that require answers, and various treatments need stronger evidence to establish their precise role and efficacy. This Research Topic aims to provide some answers to these questions.

## References

[1] McDonald P. Colorectal quotations. J R Soc Med. (2005) 98(2):77–8. 10.1177/014107680509800214 15684365 PMC1079390

[2] Pata F Sgró A Ferrara F Vigorita V Gallo G Pellino G. Anatomy, physiology and pathophysiology of haemorrhoids. Rev Recent Clin Trials. (2021) 16(1):75–80. 10.2174/1574887115666200406115150 32250229

[3] Cocorullo G Tutino R Falco N Licari L Orlando G Fontana T The non-surgical management for hemorrhoidal disease. A systematic review. G Chir. (2017) 38(1):5–14. 10.11138/gchir/2017.38.1.005 28460197 PMC5730401

[4] Pata F Gallo G Pellino G Vigorita V Podda M Di Saverio S Evolution of surgical management of hemorrhoidal disease: an historical overview. Front Surg. (2021) 8:727059. 10.3389/fsurg.2021.727059 34527700 PMC8435716

[5] Erozkan K Gorgun E. Robotic colorectal surgery and future directions. Am J Surg. (2023):S0002-9610(23)00574-3. 10.1016/j.amjsurg.2023.10.046 37953126

[6] Spinelli A Carrano FM Laino ME Andreozzi M Koleth G Hassan C Artificial intelligence in colorectal surgery: an AI-powered systematic review. Tech Coloproctol. (2023) 27(8):615–29. 10.1007/s10151-023-02772-8 36805890

[7] Picciariello A Tsarkov PV Papagni V Efetov S Markaryan DR Tulina I Classifications and clinical assessment of haemorrhoids: the proctologist’s corner. Rev Recent Clin Trials. (2021) 16(1):10–6. 10.2174/1574887115666200312163940 32164517

[8] Bracchitta S Bracchitta LM Pata F. Combined rubber band ligation with 3% polidocanol foam sclerotherapy (ScleroBanding) for the treatment of second-degree haemorrhoidal disease: a video vignette. Colorectal Dis. (2021) 23(6):1585–6. 10.1111/codi.15613 33660907

[9] Pata F Bracchitta LM D'Ambrosio G Bracchitta S. Sclerobanding (combined rubber band ligation with 3% polidocanol foam sclerotherapy) for the treatment of second- and third-degree hemorrhoidal disease: feasibility and short-term outcomes. J Clin Med. (2021) 11(1):218. 10.3390/jcm11010218 35011962 PMC8745462

[10] 2015 European Society of Coloproctology Collaborating Group. The impact of stapling technique and surgeon specialism on anastomotic failure after right-sided colorectal resection: an international multicentre, prospective audit. Colorectal Dis. (2018) 20(11):1028–40. 10.1111/codi.14308 29920945

[11] 2017 European Society of Coloproctology (ESCP) collaborating group. An international multicentre prospective audit of elective rectal cancer surgery; operative approach versus outcome, including transanal total mesorectal excision (TaTME). Colorectal Dis. (2018) 20(Suppl 6):33–46. 10.1111/codi.14376 30255642

[12] Ma L Pang X Ji G Sun H Fan Q Ma C. The impact of anastomotic leakage on oncology after curative anterior resection for rectal cancer: a systematic review and meta-analysis. Medicine (Baltimore). (2020) 99(37):e22139. 10.1097/MD.0000000000022139 32925766 PMC7489661

[13] GlobalSurg Collaborative. Surgical site infection after gastrointestinal surgery in high-income, middle-income, and low-income countries: a prospective, international, multicentre cohort study. Lancet Infect Dis. (2018) 18(5):516–25. 10.1016/S1473-3099(18)30101-4 29452941 PMC5910057

[14] GlobalSurg Collaborative. Surgical site infection after gastrointestinal surgery in children: an international, multicentre, prospective cohort study. BMJ Glob Health. (2020) 5(12):e003429. 10.1136/bmjgh-2020-003429 33272940 PMC7716674

[15] Kamboj M Childers T Sugalski J Antonelli D Bingener-Casey J Cannon J Risk of surgical site infection (SSI) following colorectal resection is higher in patients with disseminated cancer: an NCCN member cohort study. Infect Control Hosp Epidemiol. (2018) 39(5):555–62. 10.1017/ice.2018.40 29553001 PMC6707075

[16] Garoufalia Z Wexner SD. Indocyanine green fluorescence guided surgery in colorectal surgery. J Clin Med. (2023) 12(2):494. 10.3390/jcm12020494 36675423 PMC9865296

[17] Picchetto A Cinelli L Bannone E Baiocchi GL Morales-Conde S Casali L Fluorescence-based sentinel lymph node mapping and lymphography evaluation: results from the IHU-IRCAD-EAES EURO-FIGS registry. Surg Endosc. (2023) 37(7):5472–81. 10.1007/s00464-023-10043-8 37043006

[18] Picchetto A Seeliger B La Rocca S Barberio M D'Ambrosio G Marescaux J Fluoreszenzgesteuerte detektion von lymphknotenmetastasen bei gastrointestinalen tumoren [fluorescence-guided detection of lymph node metastases of gastrointestinal tumors]. Chirurg. (2019) 90(11):891–8. 10.1007/s00104-019-01039-z 31552436

[19] Johnson KB Wei WQ Weeraratne D Frisse ME Misulis K Rhee K Precision medicine, AI, and the future of personalized health care. Clin Transl Sci. (2021) 14(1):86–93. 10.1111/cts.12884 32961010 PMC7877825

